# Activation of Egr-1 in Human Lung Epithelial Cells Exposed to Silica through MAPKs Signaling Pathways

**DOI:** 10.1371/journal.pone.0068943

**Published:** 2013-07-18

**Authors:** Ling Chu, Tiansheng Wang, Yongbin Hu, Yonghong Gu, Zanshan Su, Haiying Jiang

**Affiliations:** 1 Department of Pathology, Third Xiangya hospital, Central South University, Changsha, Hunan, PR China; 2 Department of Otolaryngology, Third Xiangya hospital, Central South University, Changsha, Hunan, PR China; 3 Department of Pathology, Xiangya hospital, Central South University, Changsha, Hunan, PR China; UAE University, Faculty of Medicine & Health Sciences, United Arab Emirates

## Abstract

The alveolar type II epithelial cell, regarded historically as a key target cell in initial injury by silica, now appears to be important in both defense from lung damage as well as elaboration of chemokines and cytokines. The molecular basis for silica-induced epithelial cell injury is poorly understood. In this study we explored the activation of nuclear factor Egr-1 and related signal pathway. Human II alveolar epithelial line A549 cells were exposed to silica for indicated time to assay the expression and activation of Egr-1 and upstream MAPKs. Immunofluorescence, western-blot techniques, RT-PCR, Electrophoretic mobility shift assay (EMSA), transient transfection assay, kinase inhibitor experiments were performed. It was found that the expression of Egr-1 at mRNA and protein level was significantly increased in A549 cells after administration with silica and the activity of Egr-1 peaked by silica treatment for 60 minutes. Furthermore, phosphorylated-ERK1/2, P38 MAPKs (the upstream kinase of Egr-1) ballooned during 15-30minutes, 30-60minutes respectively after silica exposure in A549 cells. By administration of ERK1/2, P38 inhibitor, the expression and transcription of Egr-1 were both markedly decreased. But PKC inhibitor did not prevent the increase of Egr-1. These results indicated Egr-1 played a critical role in silica-induced pulmonary fibrosis in an ERK1/2, P38 MAPKs-dependent manner, which suggests Egr-1 is an essential regulator in silicosis, and underlines a new molecular mechanism for fibrosis induced by silica.

## Introduction

Tissue fibrosis, the pathological hallmark of scleroderma/systemic sclerosis, pulmonary fibrosis, glomerulosclerosis, and other chronic diseases, is a major determinant of morbidity and mortality [1]. Currently there are no effective therapies to arrest or reverse the process of fibrosis. Furthermore, despite its enormous clinical impact, the pathogenesis of this disease remains poorly understood. Recently, growing studies demonstrated Egr-1 played a key role in the pathogenesis of fibrosis [2–5]. But how Egr-1 affects the process of pulmonary fibrosis especially silicosis was seldom reported.

Silicosis is an inflammatory and fibrotic lung disease caused by inhalation and deposition of silica dust. As known, inflammation and fibrosis following silica inhalation has been associated with persistent up-regulation many “proinflammatory” factors such as TNF-alpha [6] and TGF-beta [7]. Accumulating evidence proved that transcription of the majority of these proinflammatory factors is regulated by Egr-1 [8]. Egr-1 is an 80-82 kDa-inducible zinc finger transcription factor that has also been identified as nerve growth factor-induced A, Krox-24, ZIF-268, ETR-103, and TIS-8, discovered independently by a number of laboratories searching for factors regulating cell growth and proliferation [9,10]. EGR-1 was originally identified as one immediate early gene [11]. It mediates its effects by regulating the transcription of a wide array of downstream genes involved in inflammation [12], matrix formation [13], apoptosis [14] and remodeling [15]. Increasing studies proved that Egr-1 played an important role in inflammation and fibrogenic diseases [8,12,16–18]. Grotegut et al [19] demonstrated Egr-1 induces epithelial-mesenchymal transition (EMT), an important cellular response involved in silicosis. So increasing evidence indicates Egr-1 is the key regulator in the progression of silicosis. In this paper we focus on the expression and activation of Egr-1 in A549 cells (a cell model for lung epithelial cells) exposed to silica and related signal pathway.

The signal transduction pathways leading to Egr-1 activation have been well-discussed in the last several years. In many conditions Egr-1 is a downstream target of phosphorylated MAPKs, which means that the activation of Egr-1 is dependent on MAPKs phosphorylation [20,21]. In this report we explored whether silica could induce the activation of Egr-1 in A549 cells and mediated by MAPKs. We found that silica obviously induced the expression and activation of Egr-1 and mainly mediated by ERK1/2, P38 MAPKs, but not by PKC. Our data demonstrated there was a pathway silica-ERK1/2, P38 MAPKs -Egr-1 in lung epithelial cells which might play a significant role in the pathogenesis of silicosis.

## Materials and Methods

### Silica dioxide

The silica dioxide (Sigma, St. Louis, MO, USA) was prepared by washing with HCL to remove contaminating Fe_2_O_3_ according to a method described previously [22]. Briefly, silica was boiled in 1M HCL, washed several times in water and dried in an oven at 110°C. Then particles were sterilized by heated at 160°C for 90 minutes.

### Nuclear Translocation Analysis

A549 cells were grown on chamber-slides and subjected to stimulation of silica dioxide (100µg/ml) followed by different time point (0, 30, 60, 120, 240, 480minutes) in the resting state. After being rinsed and fixed. All subsequent steps were performed in a humidified chamber at room temperature. The fixed cells were incubated with 10% serum/PBS for 20 minutes, washed with 1% BSA/PBS, and then incubated with rabbit polyclonal anti-Egr-1 antibody (2 µg/ml) (Santa Cruz Biotechnology, Santa Cruz, CA) for 1 hour. After rinsed, incubated with a fluorescent isothiocyanate (FITC)-labeled goat anti-rabbit IgG (1:200 dilution; Sigma, St. Louis, MO, USA) in the dark for 45 minutes and washed. The stained cells were visualized with an x20 Fluor objective (Nikon, Japan) with fluorescence illumination. Photographs were taken with the use of Tmax 100 ASA film (Kodak, Japan).

### Nuclear extracts

About 70–80% confluent cells were changed to RMIP1640 with 0.2% FCS for 24 hours, and then the cells were incubated for 0, 30, 60, 120, 240 and 480 minutes in RMIP1640 with 0.2% FCS with 100 µg/ml silica dioxides. Cells were washed with cold PBS, harvested by scraping, and pelleted. The cell pellets were then resuspended in 5 pellet volumes of buffer A (10 mM KCl, 20 mM HEPES, 1 mM MgCl_2_, 0.5 mM dithiothreitol, and 0.5 mM phenylmethanesulfonyl fluoride), incubated on ice for 10 minutes, and centrifuged for 10 minutes. The pellets were resuspended in buffer B (10 mM HEPES, 400 mM NaCl, 0.1 mM EDTA, 1 mM MgCl_2_, 1 mM dithiothreitol, 0.5 mM phenylmethylsulfonyl fluoride, and 15% glycerol) and incubated on ice for 30 minutes. Protein extracts were cleared by centrifugation at 4°C for 15 minutes. The supernatants containing nuclear protein were collected and stored at −80°C in aliquots. Protein concentrations were determined by protein assay (Bio-Rad, Hercules, CA ,USA).

### Reverse Transcriptase Polymerase Chain Reaction (RT-PCR) RNA Isolation

Cells were treated as previous experiments, and then were pelleted and resuspended in 1 ml of Trizol (Life Technologies, Grand Island, NY, USA), and RNA was purified following the manufacturer’s instructions. RNA was treated with DNase I (Clontech, Mountain View, CA, USA) to remove contaminant genomic DNA for 1.5 hours in the presence of RNase inhibitor, and the reaction was stopped using 10× termination mix (0.1 M EDTA, pH 8, glycogen, 1 mg/ml). The enzyme was removed by phenol-chloroform extraction, and RNA was precipitated with 2 volumes of ethanol and a 1/10 volume of sodium acetate, pH 5.2. RNA was resuspended in 20 µl of H_2_O containing the RNase inhibitor and stored at −80°C. RT was performed with an RT kit (Promega, Madison, WI, USA) following the manufacturer’s instructions. cDNA was synthesized in 20µl reaction mixtures using oligo(dT) and 1 µg of total RNA as the template. PCR amplification was performed in 0.5 µl of cDNA using gene-specific primers (Egr-1: F: AGAAGGCGATGGGTGGAGACGA, R: TGCGGATGTGGGTGGTAAGGT; β-actin F: TCACCCACACTGTGCCCATCTAC, R: GAGTACTTGCGCTCAGGAGGAG). For all PCRs, the following conditions were used: a 10-minutes denaturing step at 95°C; cycles of 1 minutes at 94°C, 45 s at 58°C, and 1 minutes at 72°C; and 10 minutes at 72°C. The PCR cycle number was optimized for each gene to prevent saturation of the reaction. PCR products were analyzed by 1.5% ethidium bromide-agarose gel electrophoresis, then scanned and quantitated for band intensities. The odds of the *Egr-1* intensity and β-actin represent relative content of *Egr-1* mRNA.

### Western-Blots Analysis

Ten micrograms of nuclear proteins or 20 micrograms of proteins were separated by 10% SDS-polyacrylamide gel electrophoresis and transferred to nitrocellulose membranes. After the transfer, membranes were blocked at room temperature for 2 h with 5% bovine serum albumin in TTBS (10 mM Tris/HCl, pH 7.5; 150 mM NaCl, and 0.05% Tween 20) and blotted with 0.5% bovine serum albumin in TTBS overnight at 4°C and antibodies at concentrations as recommended by the manufacturers. Antibodies were: Egr-1(1:200, Santa Cruz), β-tublin(1:1000, Sigma) and phosphorylated and total MAPKs, P38 (1:500, New England, Beverly, MA, USA). Levels of proteins were detected with horseradish peroxidase-linked secondary antibodies and ECL System (Cell Signaling, Beverly, MA, USA). All Western blots were repeated at least three times.

### Cell immunochemistry and immunofluorescence Analysis

A549 cells were grown on chamber-slides and subjected to stimulation of silica dioxide (100µg/ml) followed by different time point (0, 15, 30, 60, 120, 240minutes) in the resting state. After being rinsed and fixed, all subsequent steps were performed in a humidified chamber at room temperature. The fixed cells were incubated with 10% serum/PBS for 20 minutes, washed with 1% BSA/PBS, and then incubated with antibody phosphorylated MAPKs, P38 (1:200, New England, Beverly, MA, USA) for 1 hour. After rinsed and incubated with second antibody, then stained with DAB staining.

The fixed cells were incubated with 10% serum/PBS for 20 min, washed with 1% BSA/PBS, and then incubated with rabbit polyclonal anti–Egr-1 antibody (2 µg/mL, Santa Cruz Biotechnology, Inc) for 1 h. After the primary antibody was removed, the cells were gently washed with 1% BSA/PBS, incubated with a fluorescein isothiocyanate (FITC)-labeled goat anti-rabbit IgG (1:200 dilution, Sigma) in the dark for 45 min and washed with PBS. Then they were mounted in gel on a glass slide. The stained cells were visualized with an x20 Fluor objective (Nikon) with fluorescence illumination. Photographs were taken with the use of Kodak Tmax 100 ASA film.

### Electrophoretic Mobility Shift Assays (EMSAs)

Electrophoretic mobility shift assay (EMSA) was performed according to the manufacturer’s protocol (EMSA Kit, Panomics, Redwood City, CA). To perform EMSA, binding reaction mixtures contain 10µg protein of nuclear extract, 1 µg poly(dI-dC),2.0 µL of 5X Binding Buffer, 4.0 µL nuclease-free water, mix above reagents and incubate at RT for 5 minutes. Add 1.0 µL of Egr-1 probe, and incubate samples at 15°C for 30 minutes in a thermal cycler, and then run the sample on non-denature gel in 4°C, and then transfer and developed. All EMSA experiments were repeated at least three times.

### Transient transfection assay

The Egr-1-responsive promoter plasmid pEBS4 luc was provided by G Thiel (University of Saarland, Germany). 1×10^5^ cells were seeded in 24 well plate,60-70% cells were confluent and washed twice by media with no serum, transfected with 0.8µg Egr-1 reporter plasmids and 0.3µg β-gal ,followed by 100 µg/ml silica for indicated times. At the end of the incubations, cells were harvested, and the cell lysates, were assayed for luciferase activities, as described [23]. Take 20 µl lysates(The rest forβ－gal assay) adding with 100µl LARⅡ and pipetted for 10 times and assayed by luminometer quickly, got RLU (relative-luciferase-unit) results. Calculate (RLU/ beta-gal activity) to correct the luciferase values for transfection efficiency. All experiments were performed in triplicate and repeated at least twice. These results are means±SD of triplicate determinations.

### Kinase Inhibitor Experiments

Confluent cells treated as above were pretreated with U0126 (30 µM), SB230580（10µM), H7 (50µM ,1.5 hours prior ) respectively or combined for 60 minutes before silica was added and stimulated for desired minutes by 100 µg/ml silica. Thereafter, extracted RNA and nuclear protein were assayed by RT-PCR and Western blots as above referred. In this process, we replaced kinase inhibitor as negative control with Dimethylsulfoxide (DMSO). U0126, SB230580 and H7 were purchased from Calbiochem (EMD Biosciences, San Diego, CA, USA).

### Statistics

All experiments were performed 3 times. Statistical comparisons were performed using a Student’s t test for unpaired samples and a one-way ANOVA for multiple comparisons. Data from multiple experiments were averaged and expressed as mean values±SEM. Differences with p-values < 0.05 were considered statistically significant.

## Results

### Silica treatment induced the expression and activation of Egr-1 in lung epithelial cell line A549 cells

**Figure 1:**
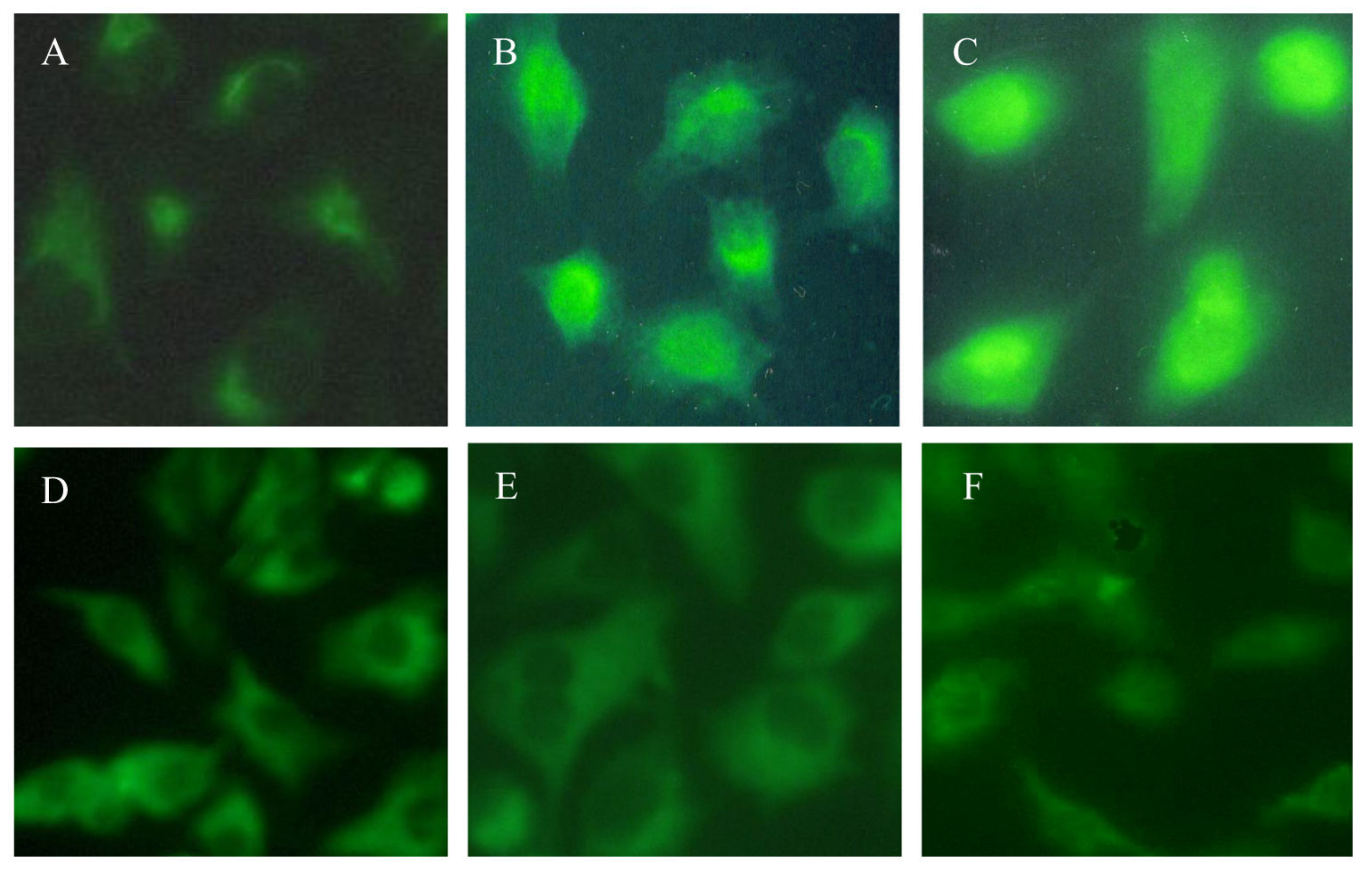
Egr-1 expression and localization in lung epithelial cell line A549. (A) 0 minute (control cells, grown in medium alone), Egr-1 expression was detected but very weak and located in cytoplasm. (B) 30 minutes, the expression of Egr-1 was increased and partly located in cytoplasm. (C) 60 minutes, robust expression of Egr-1 was located in the nuclear. (D) 120 minutes, medium expression of Egr-1 was located in cytoplasm and nuclear. (E) 240 minutes, less expression of Egr-1 was located in cytoplasm. (F) 480 minutes, Egr-1 expression almost restored to the level of control cells. Images were at ×100 magnification.

To understand the importance of Egr-1 in the pathogenesis of silica-induced diseases, studies were undertaken to determine whether silica can induce the expression and activation of Egr-1 in A549 cell lines. Immunofluorescence imaging of Egr-1 protein was performed after cells exposure to silica (100µg/ml) for 0, 30, 60, 120, 240, 480 minutes (Figure 1A–F). In resting control (0 minute), there was only weak expression of Egr-1 protein in the cytoplasm. The expression of Egr-1 appeared in nuclear after 30-minute treatment with silica, and Egr-1 protein became uniformly distributed in the nucleus after 60-minute treatment and then slowly disappeared.

### The expression of nuclear protein and mRNA of Egr-1 in A549 cells exposed to silica

**Figure 2:**
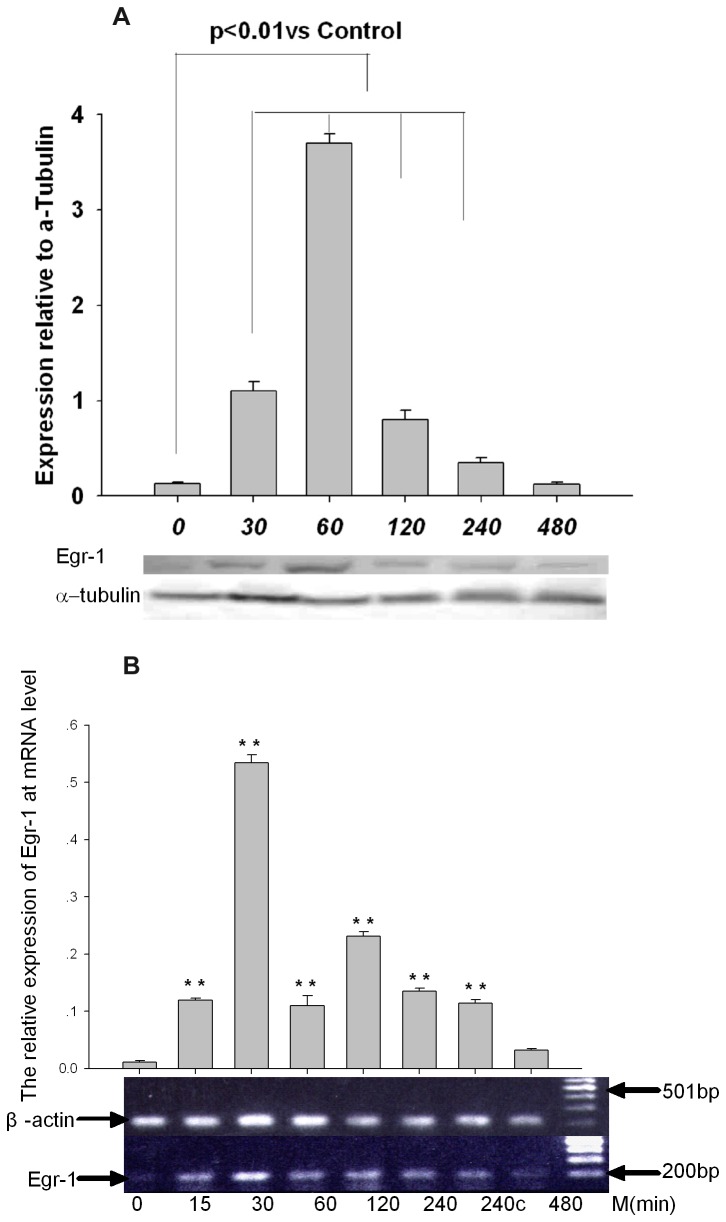
Silica induced the expression of Egr-1 at levels of nuclear protein and mRNA. (a) The dynamic expression of Egr-1 nuclear protein in A549 cells, which were exposed to silica for indicated times, was determined by western-blot using anti-Egr-1 antibody. Obvious increase of Egr-1 occurred from 30 minutes of exposure, and peaked after exposure for 60 minutes followed by gradually decrease and return to baseline by 480-minute treatment (2B). Silica induced the transcription of Egr-1 and mRNA level was determined by RT-PCR. Significant differences in Egr-1 protein and mRNA expression are noted at P<0.01(**).

The dynamic expression of Egr-1 nuclear protein was detected by western-blots in A549 cells exposure to silica and the similar results were obtained. Obvious increase of Egr-1 occurred from 30-minutes exposure, and peaked for 60-minute exposure (twenty fold more Egr-1 nuclear protein than untreated cells), followed by gradually decrease and return to baseline after 480-minute exposure (Figure 2A). The transcription of Egr-1 was detected by RT-PCR in A549 cell line, and it peaked by silica treatment for 30 minutes after exposure to silica, followed by gradual decrease and return to baseline for 480-minute treatment(Figure 2B).

### Silica increased Egr-1-DNA bining activity in A549 cells

**Figure 3:**
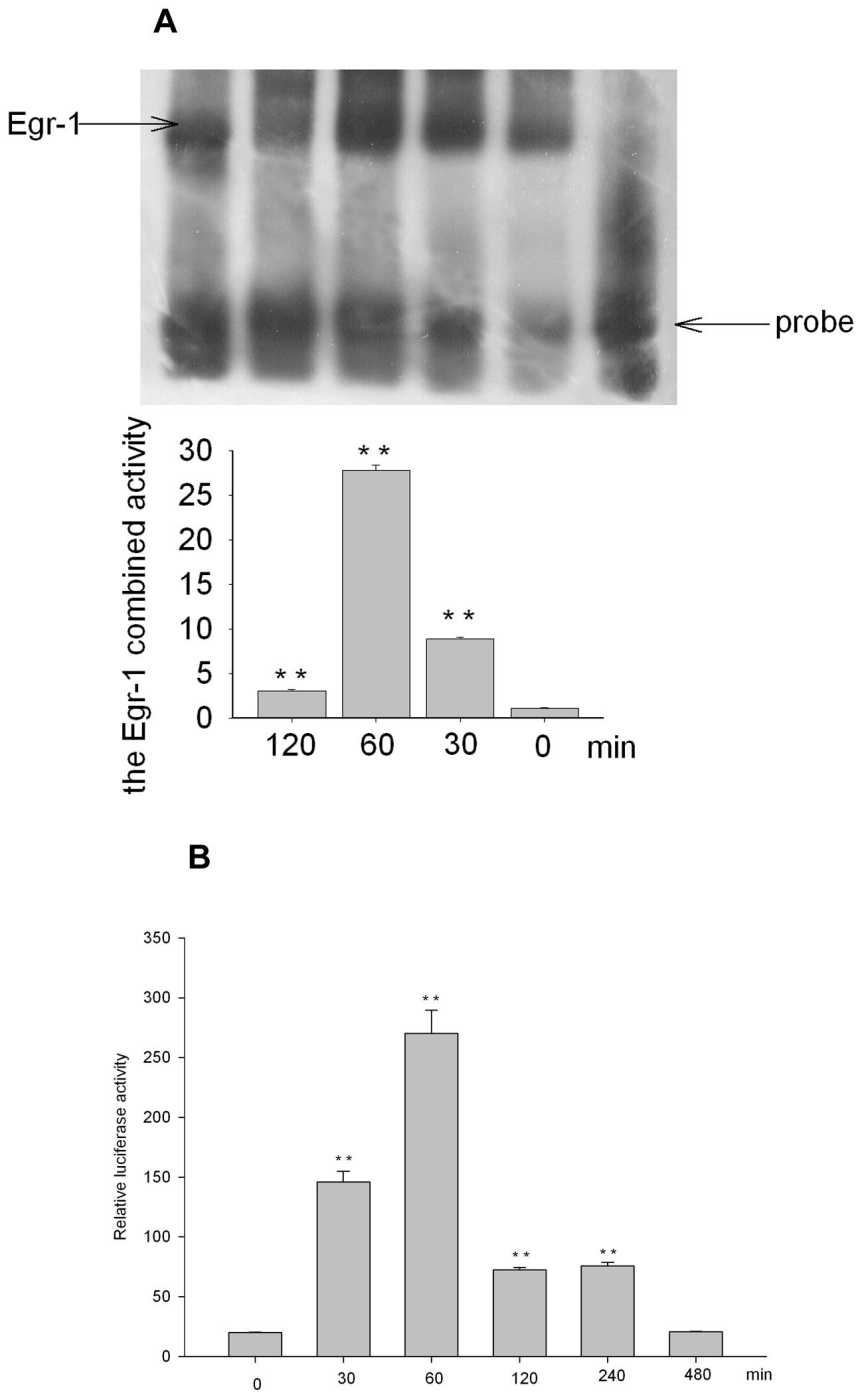
Silica increased the Egr-1 DNA binding activity in A549 cells. The binding activity of Egr-1 in A549 cells after exposure to silica was determined by EMSA experiments, and the binding activity of Egr-1 peaked after 60-minute exposure. The binding activity of Egr-1 to specific oligonucleotides probe increased from 30-minute treatment and peaked for 60-minute exposure, then decreased. And as shown in 3B: the promoter activity increased from 30-min incubation with silica and peaked at 60-min, recovered to the level of resting control till 480-min incubation. Significant differences in binding activity and luciferase activity are noted at P<0.01(**).

We examined the Egr-1-DNA bining activity in silica-treated A549 cells. The binding activity of Egr-1 in A549 cells increased from 30-minute treatment and peaked for 60-minute exposure, and then decreased after 120-minutetreatment (Figure 3A), which suggested silica could induce Egr-1 activation in A549 cells. The combining activity decreased obviously by competitor probe and antibody against Egr-1 created a supershift, which demonstrated the DNA binding activity of Egr-1 was specific (data not shown).

As shown in Figure 3B, after transfection, the cells are incubated with silica for 0-480 minutes, then harvested and cell lysates were assayed for their luciferase activities. Luciferase activities were normalized for transfection efficiency by dividing the luciferase light units by beta-galactosidase activities. And found the activities increased from 30-min incubation with silica, peaked at 60 minutes, then slowly decreased, restored to baseline till 480min.

### Silica induced ERK1/2, P38 MAPKs activation in A549 cells

**Figure 4:**
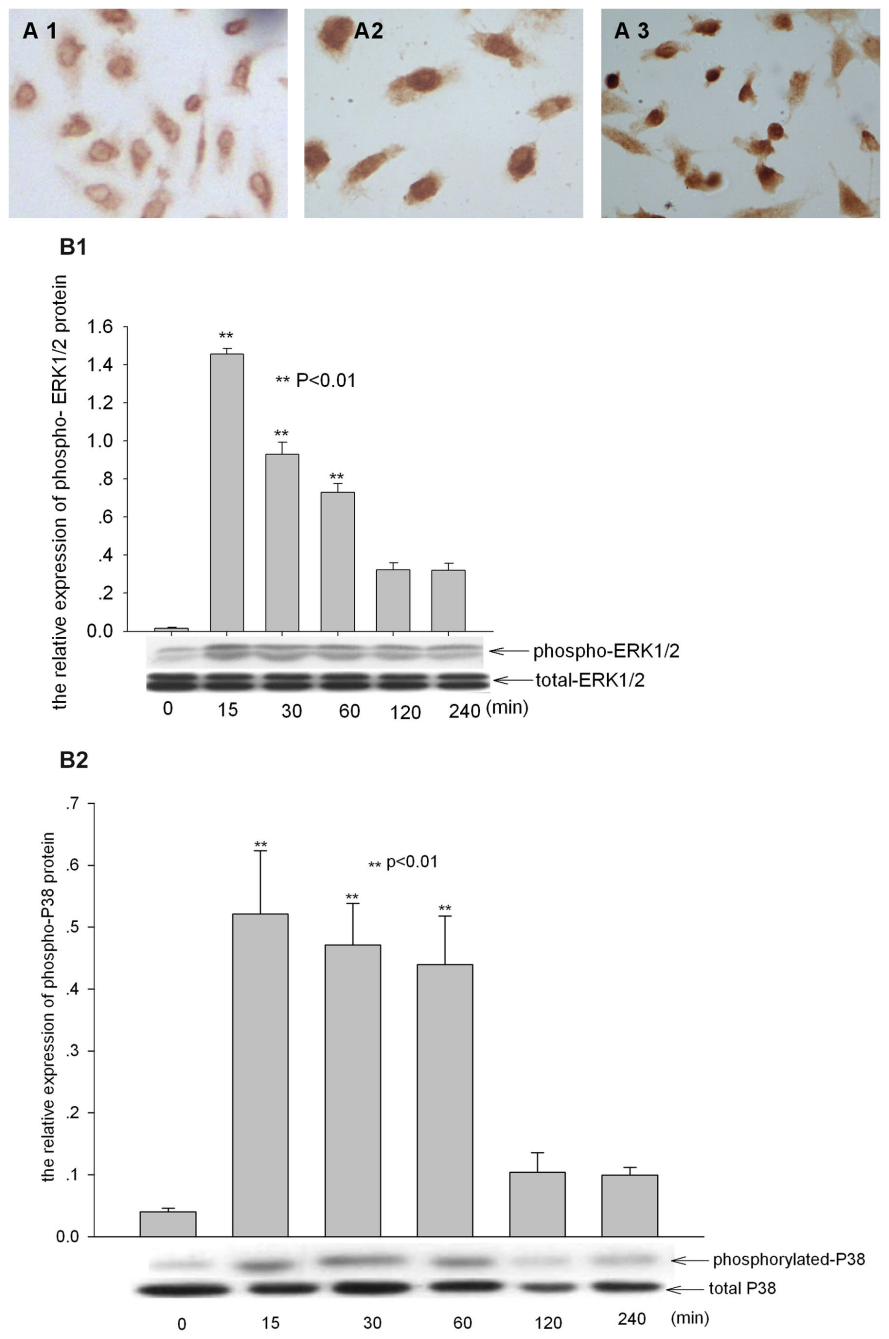
Activation of ERK1/2, P38 MAPKs in A549 cells induced by silica. (A) The level and localization of phosphorylated ERK1/2 and P38 were detected by immunochemistry. magnification: ×400. The expression of phosphorylated ERK1/2 (4A1) and P38 (picture not shown) was mainly in cytoplasm of untreated cells, and increased and located in nuclears (4A2-3) by silica treatment for 30 minutes. (B) The semi-quantitative expression of phosphorylated ERK1/2 (4B1) and P38 (4B2) in A549 cells was determined by western-blot, intensity of the bands on western blot were analyzed by Scion image and the relative expression of phosphorylated ERK1/2 and P38 to total ERK1/2 and P38 was calculated. ** p<0.01 compared to untreated cells.

We examined the expression of phosphorylated ERK1/2 and P38 MAPKs in A549 cells treated by silica through immunochemistry and western-blots. A549 cells were placed in serum-free RPMI medium for 48 hours to reduce endogenous levels of MAPKs activity, and cells were treated with 100 µg/ml silica for indicated times. The phosphorylation of MAPK p44 and p42 mainly located in cytoplasm in A549 cells unexposed to silica and mainly located in nucleus after 30 minutes exposure (Figure 4A1-2). Western-blot analysis of total cell lysates showed that silica treatment led to the phosphorylation of ERK1/2 peaked after 15-minute exposure, which returned to basal level for 240-minute treatment (Figure 4B1). Further we examined the expression and location of phosphorylated P38 in A549 cells treated with silica. And according with ERK1/2 it was also found that the phosphorylation of P38 mainly located in cytoplasm (picture not shown), and the expression of phospho-P38 mainly located in nucleus (Figure 4A3) incubated with 100 µg/ml silica, and peaked for 30-60minute treatment (Figure 4B2). Recent studies have shown that the phosphorylated MAPKs mediated the activation of Egr-1 [24–26]. Since the expression and DNA binding activity of Egr-1 peaked after 60-minute treatment, and the ERK1/2 and P38 activated after 15-30minutes exposure, a little earlier than the activation of Egr-1, we proposed that the activation of Egr-1 in A549 cells exposure to silica might be mediated by MAPKs pathway.

### Egr-1 expression and activation in epithelial cells induced by silica is dependent on the phosphorylation of MAPKs signaling

**Figure 5:**
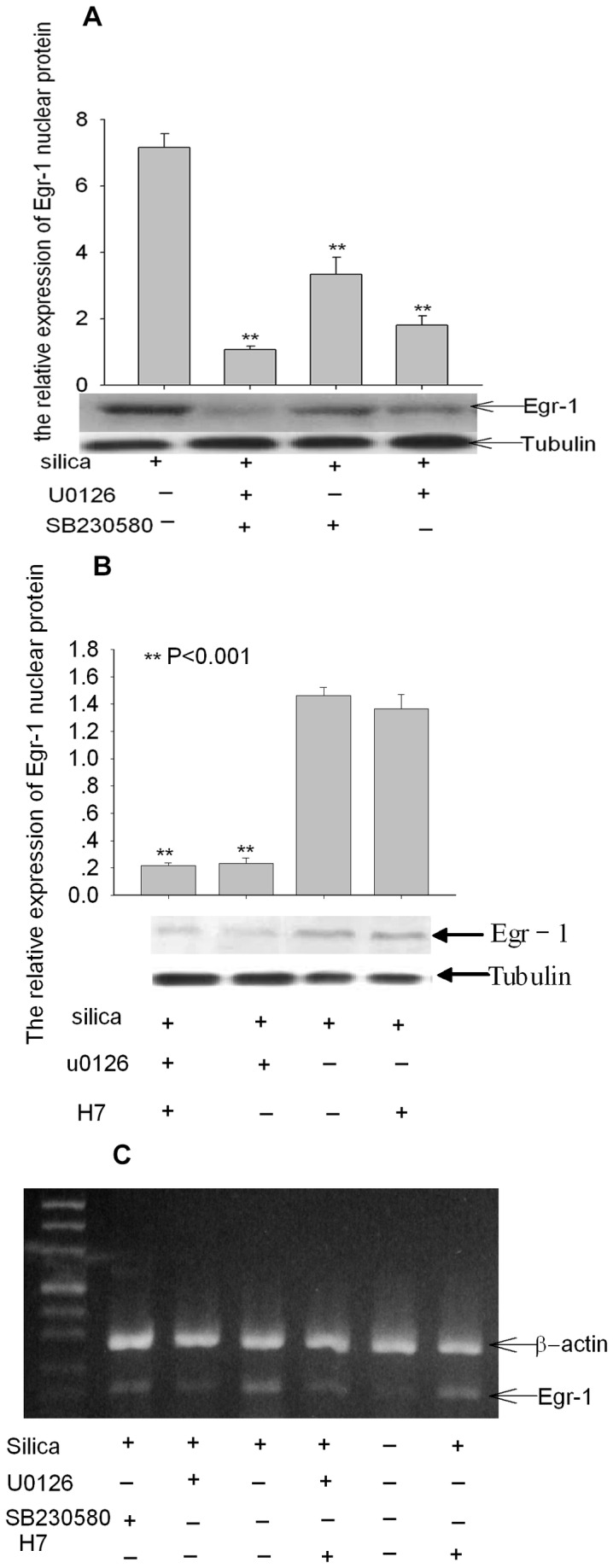
Egr-1 activation by silica was mainly dependent upon activation of the MAPKs pathway. (a) The effects of Egr-1 nuclear protein expression after administration of kinase inhibitor was determined by western-blot (5B、5C). The Egr-1 expression at nuclear protein and mRNA level was inhibited by U0126 and SB230580 respectively, but not completely disappeared by combination of both; The expression of Egr-1 was not changed by pretreatment with H7, a PKC inhibitor. The significance of Egr-1 expression is noted as P<0.05 (*), P<0.01(**).

The Egr-1 transcription factor is activated by a variety of growth factors via the MAPKs pathway [27]. We next determined whether silica-induced nuclear translocation and activation of Egr-1 was dependent upon MAPKs. The expression of Egr-1 mRNA and protein was inhibited by U0126 (an inhibitor of the ERK kinase) and SB230580（an inhibitor of the P38 kinase), which suggested that the action of silica on Egr-1 was dependent on the ERK1/2, P38 pathway. We next determined whether silica-induced nuclear translocation and activation of Egr-1 was dependent upon MAPKs. A549 cells were placed in serum-free medium for 48 h; And cells were pretreated with the MEK1/2 inhibitor U0126 (30 µM) for 60 minutes to block upstream activation of MAPKs before treatment with 100 µg/ml silica. Western-blot for phosphorylated MAPKs in cell lysates showed that this concentration of U0126 completely blocked silica mediated phosphorylation of MAPKs (data not shown); the endogenous level of phosphorylated MAPKs was also reduced. It was shown that the expression of Egr-1in nuclear protein decreased obviously, but translocation still could be seen in the presence of the MEK1/2 and P38 inhibitor (Figure 5A). While at mRNA level, MEK1/2 inhibitor U0126 and P38 inhibitor blocked the transcription of Egr-1(Figure 5C). We also applied PKC inhibitor H7 or combining H7 with U0126, and the expression of Egr-1 nuclear protein wasn’t affected by H7, which indicate PKC may not take part in this process. Taken together, these data showed that Egr-1 activation by silica was mainly dependent on activation of the ERK1/2 and P38 MAPKs pathway.

## Discussion

Crystalline silica has been shown to trigger pulmonary inflammation and fibrosis both in vivo and in vitro [28,29], but the underlying molecular mechanisms remain unclear. The alveolar type II epithelial cell, regarded historically as a key target cell in initial injury by inhaled particles, now appears to be important in both defense from lung damage as well as elaboration of chemokines and cytokines [30,31]. The molecular basis for silica-induced epithelial cell injury is poorly understood. In the present study we focused on the intracellular signaling pathways in lung epithelial cells after crystalline silica exposure.

Egr-1 is an 80–82-kD inducible zinc finger transcription factor that has also been identified as nerve growth factor–induced A, Krox-24, ZIF-268, ETR-103, and TIS-8, discovered independently by a lot of laboratories searching for factors regulating cell growth and proliferation. Increasing studies demonstrated Egr-1 played an important role in many inflammation and fibrosis diseases [20,32,33], and highly expressed in the lungs of smokers with chronic obstructive pulmonary diseases (COPD) [34]. Our study has shown elevated Egr-1 expression in silicosis in vivo (unpublished data), which indicated Egr-1 might be one of the essential factors during the pathogenesis of silicosis. According with other studies, Egr-1 expression is elicited by a large number of extracellular stimuli, typically in a rapid and transient manner. In this study, we demonstrated Egr-1 mRNA and nuclear protein were markedly induced in lung epithelial cells after exposure to silica, and reached peaks at 30 minutes and 60 minutes respectively. Reynolds et al [34] found cigarette smoke water extract could also induce the expression of Egr-1 in lung epithelial cells, which upregulated the expression of proinflammatory factors including TGF-alpha and IL-1beta. In our study, the combined activity of egr-1 with GC rich sequence also increased obviously which indicated silica could activate the nuclear factor egr-1 and activated egr-1 may play an important role when lung epithelial cells exposure to silica.

Egr-1 regulates many genes that are important for fibrogenesis. These include the profibrotic cytokines, such as TGFβ, PDGF, CTCF, VEGF, FN, PAI-1 and TIMP-1 [15]. And accumulating evidence suggests a critical role of Egr-1 in fibrosis. Elevated egr-1 mRNA or protein has been widely detected in fibrotic tissues, including fibrotic kidneys, lung tissues from patients with emphysema [35]. In accord with their study, our group(unpublished data) also found the augmented expression of Egr-1 in lung tissues from silicosis of animal model, and mainly located in pulmonary epithelial cells and macrophages. As we all known, TGFβplays an essential role during the pathogenesis of silicosis. We also found comparable expression egr-1 and TGFβprotein in lung epithelial cells in rat model of silicosis. A previous study similarly demonstrated overexpression of Egr-1 associated with increased levels of TGF-β and connective tissue growth factor in lung fibroblasts from patients with chronic obstructive pulmonary diseases [36], and other studies have shown that TGFβ induces rapid but transient expression of Egr-1 that results in stimulation of collagen gene expression [37]. Emerging studies also reveal a novel function for Egr-1 as an important mediator of TGF-β-induced responses [15]. Taken together, the activated Egr-1 might influence the progression of silicosis by TGFβ. But whether elevated TGFβ is also a potent inducer for Egr-1 in this process, in other word whether there is an Egr-1 /TGFβ positive feedback loop need to be further explored. Many studies found silica induced cell apoptosis [38,39], and recent studies [40,41] demonstrated that Egr-1 took part in regulating cell apoptosis, but whether activated Egr-1 plays an important role in silicosis by affecting cell apoptosis need further study to elucidate.

As many studies shown, depending on the stimulus and cell type, various signal transduction pathways induce the expression and activation of Egr-1, including pathways mediated by the MAP-kinase ERK1/2, protein kinase C (PKC-), RhoGTPase, or p38/c-Jun N-terminal kinase (JNK) [42]. Our study found the expression of phosphorylated ERK1/2 and P38 reached peak at 15-30minutes when cells exposure to silica and its activity also peaked, which is earlier than the egr-1 activation time. As our results shown, Egr-1 nuclear protein reached peaks at 30minutes and 60minutes respectively, and to explore whether the activation of Egr-1 depends on ERK1/2 and (or) P38 MAPKs, MAPKs inhibitor U0126 and SB230580 were used, and the expression of Egr-1 nuclear protein and mRNA decreased obviously, which suggested the expression and activation of Egr-1 in lung epithelial cells mainly mediated by ERK1/2 and P38. A recent report demonstrated that cigarette smoke and TGFβ could stimulate the activation of Egr-1 in an ERK1/2 manner in fibroblasts corroborating our findings [33]. And we also found that the expression of Egr-1 nuclear protein obviously decreased but didn’t restore to resting level by using MAPK inhibitor U0126 and SB230580, which indicated other protein kinase pathway except p38 and ERK1/2 may also involve in this process. Lyoda et al [24] demonstrated lysophosphatidic acid induced Egr-1 protein expression via PKCdelta-regulated ERK and JNK activation in vascular smooth muscle cells. Rasan et al [43] also reported that glucose-induced EGR-1 expression was mediated by PKC activation. In our study, we further applied PKC inhibitor H7 or combining H7 with U0126, and the expression of Egr-1 nuclear protein wasn’t affected by H7, which indicate PKC may not take part in this process. This is consistent with the finding [44] that LPA upregulation of Egr-1 is strictly mediated by ERK1/2 pathway, no involvement of PKC.

## Conclusions

To the best of our knowledge, this is the first report to show that egr-1 is markedly induced in epithelial cells after exposure to silica and mainly through ERK1/2, P38 MAPK pathway, which suggests Egr-1 is an essential regulator in silicosis, and underlines a new molecular mechanism for fibrosis induced by silica. This study raises the possibility that blocking excessive Egr-1 signaling might be a potential therapeutic strategy to control silicosis related disease. But how activated Egr-1 regulates silicosis? Regulating proinflammatory factors, profibrotic factors, proapoptotic genes or prompting the epithelial-to-mesenchymal transition need to be further studied.
